# Assessment and Impact of the Risk of Exposure of Portuguese Biomedical Scientists in the Context of COVID-19 †

**DOI:** 10.3390/ijerph18137097

**Published:** 2021-07-02

**Authors:** Ana Sofia R. Tavares, Fernando Bellém, Renato Abreu, Céu Leitão, Nuno Medeiros, Patrícia Alves, Luís Calmeiro

**Affiliations:** 1 H&TRC—Health & Technology Research Center, Escola Superior de Tecnologia da Saúde Instituto, Politécnico de Lisboa, 1990-096 Lisbon, Portugal; renato.abreu@estesl.ipl.pt (R.A.); mcleitao@estesl.ipl.pt (C.L.); nuno.medeiros@estesl.ipl.pt (N.M.); 2015224@alunos.estesl.ipl.pt (P.A.); 2 Escola Superior de Tecnologia da Saúde, Instituto Politécnico de Lisboa, 1990-096 Lisbon, Portugal; fernando.bellem@estesl.ipl.pt; 3 IHC—NOVA FCSH, Institute of Contemporary History, Universidade Nova de Lisboa, 1069-061 Lisbon, Portugal; 4 School of Applied Sciences, Abertay University, Dundee DD1 1HG, UK; l.calmeiro@abertay.ac.uk; 5 Faculty of Medicine, Institute of Environmental Health, University of Lisbon, 1649-028 Lisbon, Portugal

**Keywords:** risk exposure, stress appraisal, biomedical scientist, COVID-19

## Abstract

Currently, the main public health concern worldwide is the COVID-19 pandemic, caused by SARS-CoV-2, which was recently discovered and described. Due to its high pathogenicity and infectiousness, it is necessary to determine the risk healthcare professionals face every day while dealing with infected patients and contaminated biological samples. The purpose of this study was to assess Portuguese Biomedical Scientists’ risk of COVID-19 exposure and related stress appraisals. One hundred and forty participants completed online versions of the WHO’s Risk Assessment and Management of Exposure Survey and the Stress Appraisal Measure. Participants worked mainly in outpatient settings (45%), and in emergency services (28%). Twenty three percent of participants were exposed to COVID-19 through community exposure, and 39% through occupational exposure. Although 95% reported using personal protective equipment (PPE), 83.6% were at high risk of infection. However, the use of some types of PPE was related to the risk of exposure. Participants reported moderate perceptions of stress and threat, but also moderate perceptions of control over the situation. These results may contribute to a more efficient risk management of these professionals and prevent disease transmission in hospitals and communities.

## 1. Introduction

Pandemic transmission of coronavirus disease (COVID-19) due to severe respiratory syndrome coronavirus 2 (SARS-CoV-2) is a new and emerging public health concern. Coronaviruses are ribonucleic acid (RNA) viruses, widely spread among humans, other mammals, and birds [1]. They can cause multiple infections in animals and humans, especially respiratory diseases [2]. Data strongly suggest that the transmission of this virus from person to person is more frequent during close contact [3], particularly in the early stages of unsuspected infections, when viral loads tend to be high [4]. It can also occur through contact with contaminated surfaces and objects, mainly through respiratory droplets containing viral particles released by infected persons [5]. Droplets can settle on objects or surfaces that surround the infected person and infect other people when they touch these objects or surfaces with their hands, then touching their eyes, nose or mouth [5]. The incubation period for the novel coronavirus is on average 3 to 7 days [6]. Although sometimes asymptomatic, the symptoms of COVID-19 include fever, cough, sore throat, tiredness, muscle pain, anosmia, loss of taste, and, in the most severe cases, pneumonia, potentially leading to septic shock and death [5]. Evidence suggests that transmission can occur before the onset of symptoms; hence community transmission can be reduced using face masks, preventing infected persons who are asymptomatic from virus transmission [7]. According to the World Health Organization (WHO), transmission prevention measures are essential in health facilities and in the community. The most effective preventive measures include proper hand hygiene with alcohol or soap and water, avoiding touching eyes, nose, and mouth, practicing breathing etiquette, using face masks and maintaining social distance [8].

In Portugal, COVID-19 struck as early as 2 March 2020 [9]. On March 16th, the Portuguese government called a “state of emergency” and put in place a restrictive package of lockdown measures. At that time, there were no reported deaths and a record of 62 cases of COVID-19 per million inhabitants was registered, which represented a less unfavorable epidemiological scenario compared to those observed in other European countries, such as France, Italy, The United Kingdom or Spain [10,11]. The mitigation policy implemented by the government prevented a significant surge of cases with the potential to overwhelm the National Health Service (NHS). After easing restrictions in May, during the summer there was a small but moderate increase in the reproduction number (*R*_0_) [12], followed by a significant rise in October and November. A second wave emerged, forcing the adoption of new restrictive measures, including the declaration of a “state of calamity”. A sudden fast-paced rise in the number of cases and deaths in January and early February resulted in the highest number of COVID-19 cases per capita in Europe. At the time, the country registered record numbers of daily deaths (303) [13] and new positive infections (15,333) [14]. This stressed the NHS to a point of near saturation and forced Portugal to enter a second rigorous lockdown that translated into an immediate sharp decrease of COVID-19-related figures (i.e., deaths, new infections, hospitalization, ICU attendance). As of early April 2021, the disclosed numbers were among the lowest in the world, paving the way for the cessation of the “state of emergency” and the phasing out of confinement measures.

Health professionals are on the frontline of the fight against this pandemic; hence, they have one of the highest risks of infection [15]. In Portugal, it is estimated that 11% of health professionals have developed COVID-19 in their workplace [16]. As such, they may introduce or amplify outbreaks in their health units and in the community [17]. 

Frontline health professionals need their risk of exposure in their professional context thoroughly assessed to prevent the transmission of the disease [18]. According to the Center for Disease Control and Prevention (CDC) [19], healthcare professionals are at high risk of exposure to COVID-19 when they (i) have contact with a person infected by the virus in the community; (ii) provide direct support to a patient with COVID-19 (e.g., physical examinations, performing aerosol-generating procedures, sample collection, radiological examinations) without the use of adequate personal protective equipment (PPE) or proper hand hygiene, or (iii) contact with infected secretions of a COVID-19 patient or a contaminated patient care environment, without the use of adequate PPE or proper hand hygiene. Biomedical scientists are particularly prone to risk, given their proximity to the virus and disease while engaging in specimen collection and analysis. Thus, a survey of health professionals risk exposure will support a set of recommendations for the prevention and control of infection by the new Coronavirus, avoiding its spread and providing a safer working environment. 

The risk exposure in the workplace is a stressful circumstance that can affect the health and well-being of biomedical scientists. According to Lazarus [20], stress is a process that emerges because of how an individual assesses situations that are personally meaningful; such assessments are called cognitive appraisals. These can be categorized as primary appraisals and secondary appraisals. Primary appraisals refer to the extent to which the situation is central to one’s wellbeing, whether important personal goals are at stake and the level of commitment one is willing to put forth. Secondary appraisals refer to the extent to which one has the necessary resources to cope with the situation. In relation to primary appraisals, if individuals consider that there is an important goal at stake or that the situation engages core values, then a condition of stress is perceived, and threat or challenge are experienced. Threat refers to the possibility that damage to oneself may occur in the future; challenge refers to the enthusiastic and welcoming way with which people embrace and desire the oncoming struggle. Secondary appraisals refer to evaluations of what can be done about the stressful situation; hence, it represents an expectation of control over it. Cognitive appraisals influence the development of an adaptive set of emotions and behaviors to face the stressful situation. 

The purpose of the present study was to assess (i) the risk of biomedical scientists’ exposure after contact with patients infected with COVID-19 in a professional context and (ii) the cognitive appraisals that these professionals experience when facing risk of exposure. Concerning the latter, it is hypothesized that, compared with participants with low risk of exposure, those with high risk of exposure will score significantly higher in stress, threat, and uncontrollable appraisals, and significantly lower in challenge and control appraisals.

## 2. Materials and Methods

This cross-sectional study was conducted among biomedical scientists who work in Portuguese health institutions, public or private, with potential for direct or indirect exposure to patients or their secretions and biological material contaminated by SARS-CoV-2. Ethical approval (no. 03-2020) was obtained from the Ethics Board of Lisbon School of Health Technology, Polytechnic Institute of Lisbon. 

A convenience sampling procedure was used. An adequate number of participants was determined with a sample size calculator for prevalence survey [21]. It was estimated that 142 participants were needed for a maximum error in the estimation of risk of exposure to COVID-19 of 2% and a confidence interval of 95%. The prevalence was estimated based on the number of Biomedical Scientists infected with COVID-19 and registered with the Direção-Geral de Saúde (DGS), the Directorate-General for Health (7 to 13 May 2020). This number represents about 1.5% of the 7000 biomedical scientists working in health institutions in the country [22]. A total of 233 Portuguese biomedical scientists were recruited through social networks and professional associations. Data were collected between September 2020 and January 2021, via a self-administration on-line questionnaire (see Supplementary Materials), which included the WHO’s Risk Assessment and Management of Exposure Survey [23] and the Stress Appraisal Measure (SAM) [24]. Of the 233 biomedical scientists who agreed to participate, only 140 questionnaires were completed in full. The final sample had a mean age of 40.9 ± 0.9 years (female, *n* = 106, 75.7%; male = 33, 23.6%; no response, *n* = 1, 0.7%). 

### 2.1. Instruments for Data Collection

#### 2.1.1. WHO’s Risk Assessment and Management of Exposure Survey 

The WHO’s Risk Assessment and Management of Exposure Survey was translated into Portuguese using a back-to-back translation method [25]. The instrument was initially translated into Portuguese by one of the authors and verified by two independent bilingual biomedical scientists who provided suggestions. After amendments were made, the final version was translated back into English by another independent judge and compared with the original English version. These versions were deemed equivalent, and the final Portuguese version was used in this study. This survey includes questions about sociodemographic variables (see Table 1), and measures community exposure to COVID-19, occupational exposure to COVID-19 and risk categorization of healthcare workers exposed to COVID-19. 

Participants were subject to community exposure to COVID-19 if they stayed in the same household or travelled together in proximity with a confirmed COVID-19 patient. Likewise, participants were subject to occupational exposure to COVID-19 if they: (i) provided direct care to a confirmed COVID-19 patient; (ii) had face-to-face contact with a confirmed COVID-19 patient in the healthcare facility; (iii) conducted aerosol-generating procedures on confirmed COVID-19 patients (or were present during the process), or (iv) had direct contact with the patients’ environment (e.g., bed, material equipment, bathroom).

Concerning risk categorization of healthcare workers exposed to COVID-19, biomedical scientists were categorized as “high risk” or “low risk” of COVID-19 virus infection. Biomedical scientists were considered high risk for COVID-19 infection if in relation to the provision of healthcare and aerosol-generating procedures (AGPs) they did not respond “always, as recommended” to any of the following Infection Prevention and Control (IPC) procedures: (1) use of PPE such as disposable gloves, medical masks, protective visor/goggles, disposable gown and waterproof apron; (2) removing and replacing PPE according to protocol; (3) performing hand hygiene and decontaminating surfaces at least three times per day, and (4) whether the biomedical scientist had any type of accident with body fluid/respiratory secretions of a COVID-19 patient. If participants answered “always, as recommended” on all measures they were considered at “low risk” for COVID-19 virus infection.

#### 2.1.2. Stress Appraisal Measure (SAM)

The Portuguese version of SAM [26] was used in this study. SAM consists of 28 items that represent the participants’ thinking cognitive evaluations in relation to a specific situation, in this case, working in a context of exposure to COVID-19. It measures three primary cognitive appraisals (threat, challenge, and centrality), three secondary appraisals (control-by-same, control-by-others and uncontrollability) and perception of stress. Answers are given in 5-point Likert scales (1 = not at all, 2 = slightly, 3 = moderately, 4 = considerably, 5 = extremely). In this study, all scales demonstrated appropriate internal consistency, with α-Cronbach values ranging from 0.72 to 0.86, with the exception of challenge, which had a α-Cronbach of 0.57.

### 2.2. Data Analysis

Descriptive statistics were calculated for all variables in the software IBM^®^ SPSS^®^ Statistics 26.0 (Armonk, NY, EUA). Absolute and relative frequencies were used for categorical variables. To examine the relationships between demographic variables (e.g., type of institution, gender, profession), use of PPE during healthcare interaction and use of PPE during AGPs, Pearson’s chi-square tests were utilized. Alternatively, Fisher’s exact tests were used when more than 20% of the cells had expected frequencies <5. Exposure to COVID-19 virus and risk of COVID-19 infection were calculated according to WHO’s Risk Assessment and Management of Exposure Survey recommendations. Means and standard deviations were computed for cognitive appraisal variables. Pearson Product-Moment correlations were used to assess the associations between these variables. Independent *t*-tests were used to compare these variables between participants with high and low risk of infection. All results were considered significant at the 5% significance level. 

## 3. Results

### 3.1. Sociodemographic Characteristics

The sociodemographic characteristics are presented in Table 1. The most represented professions were clinical analysis (29.3%), cardio pneumology (15.0%) and radiology (31.4%). Most of the participants worked in hospital settings (80.7%), and in public healthcare institutions (79.3%). Participants worked mainly in outpatient settings (45%) and in emergency services (28%).

### 3.2. Community Exposure to COVID-19 

Concerning community exposure, 22.9% of participants reported exposure to COVID-19: 24.3% shared the same home or surrounding space with a confirmed COVID-19 patient while only 3.6% traveled in proximity (less than one meter) with a confirmed COVID-19 patient, in any kind of conveyance.

### 3.3. Occupational Exposure to COVID-19

Among the respondents, 39% were exposed to COVID-19 in a professional context (Table 2).

### 3.4. Risk Categorization of Healthcare Workers Exposed to the COVID-19 

According to the exposure risk categorization to COVID-19, 83.6% (*n* = 117) of the respondents were classified as “high risk” and 16.4% (*n* = 23) as “low risk”. Concerning gender, 84.0% (*n* = 89) of the females and 81.8% (*n* = 27) of males were classified as “high risk”.

The use of PPE as a function of the workplace was similar across settings: 94.6% (*n* = 105) of the participants in public settings and 96.4%, (*n* = 27) of the participants in private settings used PPE. Table 3 and Table 4 represent the adherence to IPC procedures during healthcare interactions and during AGPs.

Concerning the use of PPE during healthcare interactions, significant associations with risk of exposure were found for single-use gloves (*p* = 0.014), protective visor/goggles (*p* < 0.001) and disposable gowns (*p* < 0.001). Pertaining to the association between use of PPE during AGPs and risk of exposure, significant associations were found for protective visor/goggles (*p* < 0.001), disposable gowns (*p* = 0.003), and waterproof aprons (*p* < 0.001) (Table 5). We set a criterion of an absolute frequency of 10 or higher to test specific biomedical scientists’ professional roles, so that professions with a frequency lower than 10 were aggregated in the “Other” category.

### 3.5. Stress Appraisal Measure

Analysis of asymmetry and kurtosis indicate that all variables were normally distributed: skewness values ranged from 0.02 to −0.34, while kurtosis values ranged from 0.01 to −0.53, which are within the suggested range for normally distributed data (i.e., asymmetry between −2 and 2, and kurtosis between −7 and 7) [27]. All mean scores were in the moderate range, varying between 2.94 ± 0.88 for Challenge and 3.63 ± 0.68 for Controllable-by-self. There were no significant differences in cognitive appraisals between participants with high risk and low risk of exposure (Table 6). In addition, perceptions of stress were significantly associated with threat appraisals (*r* = 0.74, *p* < 0.01) and centrality (*r* = 0.74, *p* < 0.01) and uncontrollability (*r* = 0.49, *p* < 0.01).

## 4. Discussion

Frontline healthcare professionals face significant exposure to SARS-CoV-2 since they deal directly with patients and biological samples. If they do not use PPE properly or fail to perform hand hygiene correctly, healthcare professionals can become infected and transmit the virus to other patients and colleagues increasing outbreaks in health facilities [28]. Given the fact that this is a novel disease, transmission dynamics are not fully characterized yet. Understanding such dynamics will lead to the development of preventive measures to avert transmission and outbreaks, thus avoiding an overload of the health services. 

Although participants reported a high rate of use of PPE (95%), 83.6% were classified as “high risk.” This value is slightly higher than the value found by Ashinyo et al. [29], who estimated an occupational risk of 80.4% for clinical and non-clinical healthcare workers of designated COVID-19 treatment centers in Ghana.

Current results suggest that the high risk of exposure was due to direct contact with patients infected with COVID-19 or while handling biological samples. Specifically, biomedical scientists neither performed IPC procedures correctly (i.e., not always wearing all types of PPE) nor conducted hand and surfaces’ hygiene procedures as advised. 

The use of medical masks reported was lower when conducting AGPs compared to when providing healthcare to confirmed COVID-19 patients. Nonetheless, the reported use of disposable gloves, protective visor/goggles and disposable gowns was higher in AGPs. Most of the participants always performed hand hygiene before and after handling the patient, samples, doing an aseptic procedure, and touching the patient’s surroundings and biological fluids, both while providing healthcare and in AGPs. However, when it comes to decontaminating surfaces three times per day, the percentage of use of preventive strategies is higher in AGPs. These findings may be justified by the fact that the major factors for COVID-19 infection among healthcare workers are lack of understanding of the transmission mechanisms, inadequate use and availability of PPE, uncertain diagnostic criteria, unavailability of diagnostic tests and psychological stress [30].

Indeed, Nguyen et al. [31] found that although healthcare workers who were providing care for COVID-19 patients and reported inadequate use of PPE had the highest risk, and also an increased susceptibility to infection was evident even among those reporting adequate use of PPE. This suggests that healthcare workers are always at higher risk despite the appropriate use of protection. Nevertheless, the adequate use of PPE can help lower the risk they face during their working routines. In addition, Ran et al. [32] established a relationship between COVID-19 risk factors, such as a high number of hours of work and insufficient hand hygiene after contact with patients, and the infection of healthcare professionals. 

The current results suggest that the risk of exposure does not vary according to gender or profession. This is contrary to Ashinyo et al.’s [29] findings who found a statistically significant association between type of profession and risk exposure. Nevertheless, we should consider the fact that in our study only biomedical scientists answered the questionnaire as other healthcare workers were not included. The relationship between use of PPE and risk of exposure, varies according to the type of PPE. Using disposable gloves seems to lower the risk of exposure, as well as using protective visor/goggles and a disposable gown. When performing AGPs, using protective visor/goggles, disposable gowns and a waterproof apron also seem to lower the risk of exposure to COVID-19. However, these results unveil a complex reality. The risk of exposure may differ according to variables such as the specificities of the workplace (e.g., pharmacy, clinic, hospital intensive care unit). This could either hamper or promote certain types of tasks and procedures, resulting in distinct exposure to risk.

Notwithstanding that most of the surveyed biomedical scientists reported high occupational risk, the respondents’ cognitive appraisals suggest moderate perceptions of stress and threat, but also moderate perceptions of control over the situation. These moderate appraisals are likely to be the psychological outcome of an increasing level of protection provided by PPE-related measures in place during data collection. Surprisingly, cognitive appraisals did not significantly differ between participants with high or low risk of exposure. Notably though, means for threat and challenge (primary) appraisals and control-related (secondary) appraisals are in the expected direction; nevertheless, interpretations are limited by the different sample sizes between groups.

The unpredictable and contingent nature of the COVID-19 pandemic, however, may foster in the participants a sense of lack of control over the situation. On the other hand, the availability and use of appropriate protection measures may instill a sense of control. This pattern of results highlights the intricacies of the concept of risk as a multidimensional outcome of objective conditions and subjective appreciations [33,34]. Exposure and inadequate or non-use of PPE have been associated with increased infection risk [35]. An analysis of the directional relationships between risk of exposure and cognitive appraisals may help explain the extent of use of protective measures. 

The results of this study contribute to a more complete knowledge about the risk of exposure to COVID-19 that biomedical scientists face daily. Future investigations should examine the transmission’s mechanisms to control and adopt efficient preventive measures. Additionally, studies on health professionals’ knowledge and perception of COVID-19 associated risks may pave the way to the adoption of more effective, timely and appropriate psychological monitoring and follow-up measures regarding concrete work contexts and specific needs of each occupational healthcare group.

This study helps our understanding of the risk of exposure that biomedical scientists face at work. However, it has a limited number of participants, therefore, these results cannot be generalized, and further studies are needed to reach a better understanding on this matter. Another limitation of this study is that assessing occupational exposure to COVID-19 depends on the participants’ recall of different situations to which they were exposed. In addition, there is a tendency to not select extreme options such as “always as recommended” or “never”, thus these aspects may overestimate the classification considered in the study. Nevertheless, the instrument used is a standardized measure developed by WHO, which allows for direct comparisons with other studies based on samples from different cultures.

## 5. Conclusions

Most biomedical scientists are at high risk of occupational exposure to COVID-19. However, perceptions of stress and threat are moderate, possibly filtered by a general sense of safety. In fact, the risk can be reduced by consistent and appropriate use of PPE, which is reported by most of the health professionals as disposable gloves and medical masks. To be considered as “low risk of infection”, participants must use all types of PPE “always, as recommended” and failure to do so, even for one PPE, places the professional in the “high risk of infection” category [18]. However, the use of only, disposable gloves and medical masks might be enough for them to feel safe, providing an inaccurate interpretation of risk. It would be useful to ascertain the reasons for lack of compliance with the full recommendations for PPE use. Recommendations for protection rules must reinforce concrete measures of support and working conditions offered by healthcare organizations alongside the adoption of a policy encouraging the development of personal agency.

## Figures and Tables

**Table 1:** Sociodemographic characteristics of biomedical scientists.

Variable	Absolute Frequency	Percentage (%)
Gender ^¥^		
Female	106	75.7
Male	33	23.6
Biomedical Scientist Profession		
Anatomic pathology	3	2.1
Audiology	2	1.4
Environmental health	1	0.7
Cardio pneumology	21	15.0
Clinical analysis	41	29.3
Neurophysiology	1	0.7
Occupational therapy	3	2.1
Oral hygiene	1	0.7
Orthoprosthetics	1	0.7
Orthoptic	3	2.1
Pharmacy	7	5.0
Physiotherapy	10	7.1
Radiology	44	31.4
Speech therapy	2	1.4
Type of healthcare setting		
Hospital	113	80.7
Outpatient clinic	12	8.6
Primary health centre	6	4.3
Home care for patients with mild symptoms	0	0.0
Other	9	6.4
Type of institution		
Public healthcare	111	79.3
Private healthcare	28	20.0
Prefer not to answer	1	0.7

*n* = 140; ^¥^ 1 missing value.

**Table 2:** Biomedical scientists’ activities performed on COVID-19 patients in healthcare facilities.

Variable	Absolute Frequency	Percentage (%)
Direct care to a confirmed COVID-19 patient ^¥^		
Yes	79	56.8
No	44	31.7
Unknown	16	11.5
Face-to-face contact (within 1 metre) with a confirmed COVID-19 patient		
Yes	80	57.1
No	35	25.0
Uknown	25	17.9
Present when AGPs were performed on the patient		
Yes	21	15.0
No	110	78.6
Uknown	9	6.4
Direct contact with patients’ environment (e.g., bed, medical equipment, bathroom)		
Yes	68	48.6
No	54	38.6
Uknown	18	12.9

*n* = 140; ^¥^ 1 missing value.

**Table 3:** Adherence to IPC procedures during healthcare interactions.

Variable	Absolute Frequency	Percentage (%)
Wearing PPE during healthcare interaction with a confirmed COVID-19 patient		
Yes	133	95.0
No	7	5.0
If yes, how often each item of PPE was used:		
Single-use gloves		
Always, as recommended	111	79.3
Most of the time	14	10.0
Occasionally	5	3.6
Rarely	3	2.1
Medical mask		
Always, as recommended	131	93.6
Most of the time	2	1.4
Occasionally	0	0.0
Rarely	0	0.0
Face shield or goggles/protective glasses		
Always, as recommended	71	50.7
Most of the time	32	22.9
Occasionally	13	9.3
Rarely	17	12.1
Disposable gown		
Always, as recommended	87	62.1
Most of the time	27	19.3
Occasionally	10	7.1
Rarely	9	6.4
Removing and replacing PPE according to protocol ^¥^		
Always, as recommended	107	76.4
Most of the time	27	19.3
Occasionally	2	1.4
Rarely	3	2.1
Performing hand hygiene before and after touching a confirmed COVID-19 patient/sample ^¥^		
Always, as recommended	121	86.4
Most of the time	16	11.4
Occasionally	2	1.4
Rarely	0	0.0
Performing hand hygiene before and after performing any clean or aseptic procedure ^¥^		
Always, as recommended	119	85.0
Most of the time	15	10.7
Occasionally	1	0.7
Rarely	4	2.9
Performing hand hygiene after exposure to body fluid		
Always, as recommended	131	93.6
Most of the time	7	5.0
Occasionally	1	0.7
Rarely	1	0.7
Performing hand hygiene after touching the patient’s surroundings ^¥^		
Always, as recommended	109	77.9
Most of the time	24	17.1
Occasionally	3	2.1
Rarely	3	2.1
Decontamine high touch surfaces frequently (at least three times daily)		
Always, as recommended	80	57.1
Most of the time	50	35.7
Occasionally	5	3.6
Rarely	5	3.6

*n* = 140; ^¥^ 1 missing value.

**Table 4:** Adherence to IPC procedures when performing AGPs.

Variable	Absolute Frequency	Percentage (%)
Wearing PPE in AGPs to confirmed COVID-19 patient ^¥^		
Yes	121	86.4
No	18	12.9
If yes, how often each item of PPE was used:		
Single-use gloves		
Always, as recommended	116	82.9
Most of the time	4	2.9
Occasionally	0	0.0
Rarely	1	0.7
Medical mask		
Always, as recommended	109	77.9
Most of the time	6	4.3
Occasionally	4	2.9
Rarely	2	1.4
Face shield or goggles/protective glasses		
Always, as recommended	80	57.1
Most of the time	15	10.7
Occasionally	11	7.9
Rarely	15	10.7
Disposable gown		
Always, as recommended	94	67.1
Most of the time	13	9.3
Occasionally	11	7.9
Rarely	3	2.1
Waterproof apron ^¥^		
Always, as recommended	48	34.3
Most of the time	17	12.1
Occasionally	22	15.7
Rarely	33	23.6
Removing and replacing PPE according to protocol ^¥¥^		
Always, as recommended	108	77.1
Most of the time	20	14.3
Occasionally	3	2.1
Rarely	5	3.6
Performing hand hygiene before and after touching a confirmed COVID-19 patient/sample ^¥¥^		
Always, as recommended	115	82.1
Most of the time	17	12.1
Occasionally	1	0.7
Rarely	4	2.9
Performing hand hygiene before and after performing any clean or aseptic procedure ^¥¥^		
Always, as recommended	119	85.0
Most of the time	14	10.0
Occasionally	0	0.0
Rarely	3	2.1
Performing hand hygiene after touching the patient’s surroundings ^¥¥^		
Always, as recommended	109	77.9
Most of the time	25	17.9
Occasionally	0	0.0
Rarely	3	2.1
Decontamine high touch surfaces frequently (at least three times daily) ^¥¥^		
Always, as recommended	95	67.9
Most of the time	32	22.9
Occasionally	5	3.6
Rarely	5	3.6

*n* = 140; ^¥^ 1 missing value; ^¥¥^ More than 1 missing value.

**Table 5:** Association between variables and risk of COVID-19.

Variable	Risk of COVID-19 Infection		*p*-Value
	Low Risk (*n* = 23)	High Risk (*n* = 116)	
	*N* (%)	*N* (%)	
Type of institution			
Public healthcare	19 (17.1)	92 (82.9)	
Private healthcare	4 (14.3)	24 (85.7)	
Gender			
Female	17 (16.0)	86 (84.0)	0.772 †
Male	6 (18.2)	27 (81.8)	
Profession			
Clinical analysis	7 (17.1)	34 (82.9)	0.383 †
Cardiopneumology	6 (28.6)	15 (71.4)	
Radiology	6 (13.6)	38 (86.4)	
Other	4 (11.8)	30 (88.2)	
Use of PPE in healthcare interaction			
Yes	23 (17.3)	110 (82.7)	0.599 ‡
No	0 (0.0)	7 (100.0)	
Single-use gloves	23 (17.3)	110 (82.7)	0.014 ‡ **
Medical mask	23 (17.3)	110 (82.7)	1.000 ‡
Protective visor/goggles	23 (17.3)	110 (82.7)	0.000 † ***
Disposable gown	23 (17.3)	110 (82.7)	0.000 † ***
Use of PPE in AGPs ^¥^			
Yes	22 (18.2)	99 (81.8)	
No	0 (0.0)	18 (100.0)	
Single-use gloves	22 (18.2)	99 (81.8)	0.583 ‡
Medical mask	22 (18.2)	99 (81.8)	0.121 ‡
Protective visor/goggles	22 (18.2)	99 (81.8)	0.000 † ***
Disposable gown	22 (18.2)	99 (81.8)	0.003 ‡ **
Waterproof apron	22 (18.2)	99 (81.8)	0.000 † ***
Occupational accidents ^¥^			
Yes	0 (0.0)	5 (100.0)	0.590 ‡
No	23 (17.2)	111 (82.8)	

^¥^ 1 missing value. † Significant associations were assessed using Pearson’s chi-square test. ‡ Significant associations were assessed using Fisher’s exact tests. * *p* < 0.05, ** *p* < 0.01, *** *p* < 0.001

**Table 6:** Cognitive appraisals and stress perceptions (means and standard deviations) of biomedical scientists as a function of risk exposure to patients with COVID-19.

Variables	All	Risk		*t*-Value	*p*-Value
		Low (*n* = 23)	High (*n* = 115)		
Threat	3.10 (0.88)	3.01 (1.01)	3.12 (0.86)	−0.58	59
Challenge	2.94 (0.88)	3.14 (0.99)	2.90 (0.86)	1.20	23
Centrality	3.31 (0.91)	3.29 (1.11)	3.32 (0.88)	−0.12	90
Uncontrollability	2.69 (0.82)	2.80 (0.91)	2.67 (0.80)	0.65	52
Controllable-by-others	3.02 (0.93)	3.20 (0.84)	2.98 (0.94)	1.02	31
Controllable-by-self	3.63 (0.68)	3.73 (0.62)	3.60 (0.69)	0.80	42
Stress	3.40 (0.81)	3.41 (0.78)	3.39 (0.82)	0.10	92

## Data Availability

The data presented in this study are available on request from the corresponding author.

## Supplementary Materials

The following are available online at https://www.mdpi.com/article/10.3390/ijerph18137097/s1, General anonymous questionnaire: Questionário de Avaliação, Gestão e Impacto do Risco de Exposição dos Técnicos Superiores de Diagnóstico e Terapêutica no Contexto da COVID-19 [Questionnaire for Assessment, Management, and Impact of the Exposure Risk of Biomedical Scientists in the Context of COVID-19].
