# Supplementary material for: Assessment and Impact of the Risk of Exposure of Portuguese Biomedical Scientists in the Context of COVID-19
Source: Int J Environ Res Public Health. 2021 Jul 2;18(13):7097. doi: 10.3390/ijerph18137097 (PMC8296870; doi:10.3390/ijerph18137097)
Supplement: Supplementary file 1 [file ijerph-18-07097-s001.zip › ijerph-1222669-supplementary.pdf]

Avaliação, Gestão e Impacto do Risco de Exposição dos Técnicos Superiores de Diagnóstico e Terapêutica no Contexto da COVID-19

O presente questionário é composto por um conjunto de perguntas que pretendem avaliar o risco de exposição dos TSDT a pacientes confirmados com a COVID-19. Por favor, leia cada pergunta com atenção e assinale a sua resposta. Não há respostas certas ou erradas. A sua colaboração é muito importante neste estudo, uma vez que permitirá gerar conhecimento acerca desta problemática, por forma a suportar um conjunto de recomendações para a gestão do risco e proteção da saúde destes profissionais, no desempenho das suas funções.

Obrigado

TERMO DE CONSENTIMENTO LIVRE E INFORMADO
Informação Geral: É convidado/a participar nesta pesquisa, no âmbito de um Projeto de Investigação acerca do risco de exposição e impacto nos Técnicos Superiores de Diagnóstico e Terapêutica no contexto da COVID-19, a decorrer na Escola Superior de Tecnologia da Saúde de Lisboa, do Instituto Politécnico de Lisboa (ESTeSL/IPL) e o Centro de Investigação em Saúde e Tecnologia (H&TRC). É importante que leia a informação seguinte, antes de concordar em participar nesta pesquisa. Este texto descreve, de forma sucinta, a pesquisa, os seus objetivos gerais e o que se espera da sua participação, incluindo a identificação dos procedimentos de recolha de dados, riscos previstos, os seus direitos e confidencialidade dos dados. Porque é convidado a participar nesta pesquisa? Este estudo pretende investigar, o risco de exposição e o impacto do mesmo, nos TSDT que trabalhem em instituições de saúde portuguesas, públicas ou privadas, com potencial de exposição direta ou indireta a pacientes ou a suas secreções e material biológico contaminado pelo vírus da COVID-19. Abandono da pesquisa sem qualquer penalização A sua participação é voluntária e pode recusar-se a participar. Caso decida participar neste estudo, poderá desistir a qualquer momento, sem qualquer tipo de consequência. A recusa ou desistência não afetará a sua relação com a ESTeSL/H&TRC nem com os sindicatos onde foi recrutado. Explicação do procedimento Caso aceite fazer parte deste estudo, deverá clicar na caixa de resposta que diz "PRETENDO participar nesta investigação", por forma a respeitar os padrões éticos da pesquisa online e dar o seu consentimento. Procedimentos de recolha de dados A recolha de dados será feita em dois momentos: num primeiro momento ser-lhe-á solicitado o preenchimento de um questionário online, cuja duração estimada de preenchimento é cerca de 15 minutos. O segundo momento ocorrerá três meses depois e consistirá no preenchimento de outro questionário que demorará cerca de 5 minutos. Após preenchimento dos questionários, estes não deverão ser submetidos mais do que uma vez. Quais os possíveis benefícios da sua participação? Ao fazer parte deste estudo, irá dar um contributo importante para o conhecimento do risco de exposição dos TSDT, em contexto profissional, por forma a prevenir a transmissão da doença, e compreender o impacto dessa exposição, participando no avanço do conhecimento nesta área. Quais os possíveis riscos da sua participação? Devido ao teor das questões colocadas, não está previsto qualquer risco da sua participação. Confidencialidade dos dados e anonimato Todos os dados recolhidos serão confidenciais e a sua participação anónima. No entanto, no final do primeiro momento de avaliação, ser-lhe-á pedido para introduzir o seu e-mail para que, três meses depois, possa participar no segundo momento de avaliação. Para isso, os participantes serão identificados apenas com um número, que serve para o investigador ter registo da sequência pela qual o questionário foi aplicado. É impossível identificar o IP do computador ou aparelho aquando o preenchimento do questionário. Se preferir, pode participar apenas na primeira parte do estudo, não tendo necessidade de providenciar o seu e-mail. No

☐ PRETENDO participar nesta investigação
☐ NÃO PRETENDO participar nesta investigação

entanto a segunda parte do estudo é igualmente importante e complementa a primeira parte. O que acontecerá aos dados quando a investigação terminar? Os resultados da investigação serão apresentados num relatório final, podendo, se desejar, contactar a coordenadora do projeto, Ana Tavares, para se inteirar dos resultados obtidos.
Como irão os resultados do estudo ser divulgados e com que finalidades? Os resultados serão divulgados em publicações científicas, cuja finalidade é aumentar o conhecimento na área.
Em caso de dúvidas quem devo contactar? Para esclarecimentos adicionais ou questões relacionadas com esta pesquisa poderá contactar a investigadora responsável por e-mail - ana.tavares@estesl.ipl.pt

A - Informação sobre o TSDT

1 Partilha ou partilhou o mesmo domicílio ou espaço envolvente com um paciente com COVID-19?
- ☐ Sim
- ☐ Não

2 Viaja ou viajou em proximidade (menos de um metro) com um paciente com COVID-19 confirmada, em qualquer tipo de transporte?
- ☐ Sim
- ☐ Não

3 Idade ______________________________

4 Sexo
- ☐ Masculino
- ☐ Feminino
- ☐ Outro
- ☐ Prefiro não responder

5 Indique qual a sua profissão na área das Tecnologias da Saúde:
- ☐ Técnico Superior de Radiologia
- ☐ Técnico Superior de Ortóptica
- ☐ Fisioterapeuta
- ☐ Técnico Superior de Farmácia
- ☐ Técnico Superior de Análises Clínicas e de Saúde Pública
- ☐ Técnico Superior de Anatomia Patológica, Citológica e Tanatológica
- ☐ Técnico Superior de Audiologia
- ☐ Técnico Superior de Cardiopneumologia
- ☐ Higienista oral
- ☐ Técnico Superior de Medicina Nuclear
- ☐ Técnico Superior de Neurofisiologia
- ☐ Ortoprostésico/Ortoptista
- ☐ Técnico Superior de Prótese Dentária
- ☐ Técnico Superior de Saúde Ambiental
- ☐ Terapeuta Superior da Fala
- ☐ Terapeuta ocupacional
- ☐ Outra

Especifique: ______________________________

6 Indique o tipo de serviço de Unidade de Saúde em que desempenha a sua atividade profissional. Assinale todas as que se aplicam:
- ☐ Ambulatório
- ☐ Emergência
- ☐ Unidades médicas
- ☐ Cuidados de saúde intensiva
- ☐ Laboratório
- ☐ Farmácia
- ☐ Outra

Especifique: ______________________________

B - Informação relativa à interação com o paciente COVID-19.

1 Em que data esteve exposto/a a um paciente com COVID-19 confirmada (Indique, pelo menos, mês e ano, caso não saiba o dia coloque o valor "zero" no campo do dia)? Introduzir = dia/mês/ano (Ex. 00/12/2020) __________________________________

2 Indique o tipo de Unidade de Saúde em que desempenha as suas funções:
- ☐ Hospital
- ☐ Clínica ambulatória
- ☐ Centro de saúde
- ☐ Cuidados domiciliários para sintomas ligeiros
- ☐ Outra

Especifique: __________________________________

3 Indique se o tipo de serviço de saúde onde desempenha a sua atividade profissional principal pertence ao setor público ou privado?
- ☐ Setor público
- ☐ Setor privado
- ☐ Prefiro não responder

4 No seu local de trabalho, lida com múltiplos pacientes com COVID-19?
- ☐ Sim
- ☐ Não
- ☐ Não sei

Número de pacientes (indique o número médio aproximado de pacientes com que lida mensalmente, se o número exato for desconhecido): __________________________________

C - Atividades desempenhadas pelos TSDT com o paciente com COVID-19 no estabelecimento de saúde.

1 Prestou diretamente algum cuidado de saúde a um paciente com COVID-19 confirmada?
- ☐ Sim
- ☐ Não
- ☐ Não sei

2 Contactou cara-a-cara (menos de um metro de distância) com um paciente confirmado com COVID-19 no seu local de trabalho?
- ☐ Sim
- ☐ Não
- ☐ Não sei

3 Estava presente quando foram realizados no paciente procedimentos de produção de aerossóis?
- ☐ Sim
- ☐ Não
- ☐ Não sei

Que tipo de procedimentos?
- ☐ Intubação traqueal
- ☐ Tratamento nebulizador
- ☐ Sucção das vias aéreas
- ☐ Recolha de expetoração
- ☐ Traqueotomia
- ☐ Broncoscopia
- ☐ Ressuscitação cardiopulmonar (RCR)
- ☐ Outra

Especifique: __________________________________

4 Teve contacto direto com o meio físico onde o paciente com COVID-19 recebeu cuidados de saúde (por exemplo, cama, lençóis, equipamento médico, casa de banho, etc.)?
- ☐ Sim
- ☐ Não
- ☐ Não sei

5 Interagiu (de forma remunerada ou não) com um paciente confirmado COVID-19, em qualquer outra unidade de cuidados de saúde durante o período acima indicado?
- ☐ Outra unidade de cuidados de saúde (pública ou privada)
- ☐ Ambulância
- ☐ Cuidados domiciliários
- ☐ Em nenhuma outra unidade de cuidados de saúde

D - Adesão aos procedimentos de controlo e prevenção de infeção (CPI) durante a prestação de cuidados de saúde ou exames de diagnóstico e terapêutica a doentes com COVID-19.

Nas perguntas que se seguem, quantifique, por favor, a frequência com que usou Equipamento de Proteção Individual (EPI), de acordo com o recomendado: "Sempre" significa mais do que 95% do tempo; "A maioria das vezes" significa 50% ou mais, mas menos do que 100% do tempo; "Ocasionalmente" significa de 20% a 50% do tempo; "Raramente" significa menos de 20% do tempo.

1 Durante a prestação de cuidados de saúde ou exames de diagnóstico e terapêutica a um paciente/amostra COVID-19, usou equipamento de proteção individual (EPI)?

☐ Sim
☐ Não

	Sempre	A maioria das vezes	Ocasionalmente	Raramente
Para cada um dos seguintes EPI, indique a frequência com que o utilizou. Luvas descartáveis (uso único)	☐	☐	☐	☐
Máscara médica	☐	☐	☐	☐
Viseira de proteção/óculos de proteção	☐	☐	☐	☐
Bata descartável	☐	☐	☐	☐

2 Durante a prestação de cuidados de saúde ou exames de diagnóstico e terapêutica a um paciente/amostra COVID-19, removeu e substituiu o EPI de acordo com o protocolado (por exemplo, quando a máscara ficou molhada, deitou-a fora no lixo apropriado, desinfetou as mãos, etc.)?

☐ Sempre
☐ A maioria das vezes
☐ Ocasionalmente
☐ Raramente

3 Durante a prestação de cuidados de saúde ou exames de diagnóstico e terapêutica a um paciente/amostra COVID-19, higienizou/desinfetou/lavou as mãos antes e depois de tocar/manipular o paciente/amostra (independentemente do uso de luvas)?

☐ Sempre
☐ A maioria das vezes
☐ Ocasionalmente
☐ Raramente

4 Durante a prestação de cuidados de saúde ou exames de diagnóstico e terapêutica a um paciente COVID-19, higienizou/desinfetou/lavou as mãos antes e depois de realizar algum procedimento de limpeza ou assepsia (por exemplo, inserção de um cateter vascular periférico, cateter urinário, intubação, colheita de sangue, etc.)?

☐ Sempre
☐ A maioria das vezes
☐ Ocasionalmente
☐ Raramente

5 Durante a prestação de cuidados de saúde ou exames de diagnóstico e terapêutica a um paciente COVID-19, desinfetou as mãos depois da exposição a fluidos orgânicos?

☐ Sempre
☐ A maioria das vezes
☐ Ocasionalmente
☐ Raramente

6 Durante a prestação de cuidados de saúde ou exames de diagnóstico e terapêutica a um paciente COVID-19, desinfetou as mãos depois de tocar o envolvimento físico imediato do paciente (cama, maçaneta da porta, bancada, telefone,etc.), independentemente do uso de luvas?

☐ Sempre
☐ A maioria das vezes
☐ Ocasionalmente
☐ Raramente

7 Durante a prestação de cuidados de saúde ou exames de diagnóstico e terapêutica a um paciente/amostra COVID-19, as superfícies de contacto foram descontaminadas frequentemente (pelos menos três vezes por dia)?

- ☐ Sempre
- ☐ A maioria das vezes
- ☐ Ocasionalmente
- ☐ Raramente

E - Adesão às medidas de controlo e prevenção de infeção (CPI) aquando a realização de procedimentos que produzem aerossóis (Intubação traqueal, tratamento de nebulização, sucção das vias aéreas, recolha de expetoração, traqueotomia, broncoscopia, ressuscitação cardiopulmonar (RCR), colheitas de sangue e outros produtos biológicos, esfregaços de sangue/expetoração, etc.).

Nas perguntas que se seguem, quantifique, por favor, a frequência com que usou Equipamento de Proteção Individual (EPI), de acordo com o recomendado: "Sempre" significa mais do que 95% do tempo; "A maioria das vezes" significa 50% ou mais, mas menos do que 95% do tempo; "Ocasionalmente" significa de 20% a 50% do tempo; "Raramente" significa menos de 20% do tempo.

1 Durante a realização de procedimentos geradores de aerossóis a um paciente/amostra COVID-19, usou EPI?

- ☐ Sim
- ☐ Não

	Sempre	A maioria das vezes	Ocasionalmente	Raramente
Luvas descartáveis (uso único)	☐	☐	☐	☐
Máscaras N95 (ou equivalente)	☐	☐	☐	☐
Viseira de proteção/óculos de proteção	☐	☐	☐	☐
Bata descartável	☐	☐	☐	☐
Avental à prova de água	☐	☐	☐	☐

2 Durante a realização de procedimentos produtores de aerossóis a um paciente/amostra COVID-19, removeu e substituiu o seu EPI de acordo com o protocolado (por exemplo, quando a máscara ficou molhada, deitou-a fora no lixo apropriado, desinfetou as mãos, etc.)?

- ☐ Sempre
- ☐ A maioria das vezes
- ☐ Ocasionalmente
- ☐ Raramente

3 Durante a realização de procedimentos produtores de aerossóis a um paciente/amostra COVID-19, desinfetou as mãos antes e depois de tocar/manipular o paciente/amostra COVID-19 (independentemente do uso de luvas)?

- ☐ Sempre
- ☐ A maioria das vezes
- ☐ Ocasionalmente
- ☐ Raramente

4 Durante a realização de procedimentos produtores de aerossóis a um paciente COVID-19, desinfetou as mãos antes e depois da realização de procedimentos de limpeza ou assepsia?

- ☐ Sempre
- ☐ A maioria das vezes
- ☐ Ocasionalmente
- ☐ Raramente

5 Durante a realização de procedimentos produtores de aerossóis a um paciente COVID-19, desinfetou as mãos depois de tocar o envolvimento físico imediato do paciente (cama, maçaneta da porta, etc.) independentemente do uso de luvas?

- ☐ Sempre
- ☐ A maioria das vezes
- ☐ Ocasionalmente
- ☐ Raramente

6 Durante a realização de procedimentos produtores de aerossóis a um paciente/amostra COVID-19, as superfícies de contacto foram descontaminadas frequentemente (pelos menos três vezes por dia)?

- ☐ Sempre
- ☐ A maioria das vezes
- ☐ Ocasionalmente
- ☐ Raramente

F - Acidentes com material biológico.

1	Durante a prestação de cuidados de saúde a um paciente COVID-19, teve qualquer tipo de acidente com fluidos orgânicos/secreções respiratórias?	☐ Sim<br>☐ Não
	Que tipo de acidente?	☐ Respingos de fluidos biológicos/secreções respiratórias nas membranas mucosas dos olhos<br>☐ Respingos de fluidos biológicos/secreções respiratórias nas membranas mucosas da boca/nariz<br>☐ Respingos de fluidos biológicos/secreções respiratórias na pele não-intacta.<br>☐ Perfuração/acidente cortante com qualquer material com fluidos biológicos/secreções respiratórias.

G - Nas perguntas seguintes procuramos saber o que pensa sobre o risco de exposição a um paciente confirmado com COVID-19. Não existem respostas certas ou erradas. Por favor, responda de acordo com a sua perceção ATUAL dessa situação. Por favor, responda a TODAS as questões. Responda a cada questão selecionando o número que melhor representa a sua resposta, de acordo com a escala seguinte:

		De modo nenhum	Ligeiramente	Moderadamente	Consideravelmente	Extremamente
1	Esta é uma situação totalmente desesperada?	☐	☐	☐	☐	☐
2	Esta situação provoca-me tensão?	☐	☐	☐	☐	☐
3	O resultado desta situação não é controlável para quem quer que seja?	☐	☐	☐	☐	☐
4	Existe alguém ou alguma organização a quem eu possa recorrer se precisar de ajuda?	☐	☐	☐	☐	☐
5	Esta situação faz sentir-me ansioso/a?	☐	☐	☐	☐	☐
6	Esta situação tem consequências importantes para mim?	☐	☐	☐	☐	☐
7	Esta situação vai ter um impacto positivo em mim?	☐	☐	☐	☐	☐
8	Quero lidar com este problema?	☐	☐	☐	☐	☐
9	O resultado desta situação poderá afetar-me?	☐	☐	☐	☐	☐
10	Esta situação pode tornar-me uma pessoa mais forte?	☐	☐	☐	☐	☐
11	O resultado desta situação será negativo?	☐	☐	☐	☐	☐
12	Tenho a capacidade para me sair bem nesta situação?	☐	☐	☐	☐	☐
13	Esta situação tem implicações sérias para mim?	☐	☐	☐	☐	☐
14	Tenho o que é preciso para me sair bem nesta situação?	☐	☐	☐	☐	☐
15	Tenho ajuda disponível para lidar com este problema?	☐	☐	☐	☐	☐
16	Esta situação sobrecarrega ou excede as minhas capacidades de resposta?	☐	☐	☐	☐	☐
17	Os recursos disponíveis são suficientes para me ajudar a lidar com esta situação?	☐	☐	☐	☐	☐
18						

	Está para além da capacidade humana poder-se fazer algo sobre esta situação?	☐	☐	☐	☐	☐
19	Fico entusiasmado/a ao pensar no desfecho desta situação?	☐	☐	☐	☐	☐
20	Esta situação é ameaçadora?	☐	☐	☐	☐	☐
21	Ninguém tem capacidade de resolver o problema?	☐	☐	☐	☐	☐
22	Serei capaz de superar este problema?	☐	☐	☐	☐	☐
23	Existe alguém que me possa ajudar a gerir esta situação?	☐	☐	☐	☐	☐
24	Considero esta situação stressante?	☐	☐	☐	☐	☐
25	Tenho as competências necessárias para ser bem-sucedido/a nesta situação?	☐	☐	☐	☐	☐
26	Lidar com este acontecimento requer esforço da minha parte?	☐	☐	☐	☐	☐
27	Esta situação tem consequências a longo prazo para mim?	☐	☐	☐	☐	☐
28	Esta situação terá um impacto negativo em mim?	☐	☐	☐	☐	☐

H - Receber os resultados por email

Se pretender receber o resultado do seu nível de risco de exposição profissional à COVID-19 deve indicar a seguir um email para receção dos resultados: ________________________

I - Participar no segundo momento de avaliação

Se pretender participar no segundo momento de avaliação do risco de exposição profissional à COVID-19 deve indicar a seguir um email para que possa ser contactado: ________________________
